# Random number datasets generated from statistical analysis of randomly sampled GSM recharge cards

**DOI:** 10.1016/j.dib.2016.12.003

**Published:** 2016-12-09

**Authors:** Hilary I. Okagbue, Abiodun A. Opanuga, Pelumi E. Oguntunde, Paulinus O. Ugwoke

**Affiliations:** a Department of Mathematics, Covenant University, Ota, Nigeria; b Department of Computer Science, University of Nigeria, Nsukka, Nigeria; c Digital Bridge Institute, International Centre for Information & Communications, Technology Studies, Abuja, Nigeria

**Keywords:** Chi-square tests, GSM recharge cards, Random number tables, Randomness

## Abstract

In this article, a random number of datasets was generated from random samples of used GSM (Global Systems for Mobile Communications) recharge cards. Statistical analyses were performed to refine the raw data to random number datasets arranged in table. A detailed description of the method and relevant tests of randomness were also discussed.

**Specifications Table**

**Table:** 

Subject area	Statistics
More specific subject area	Random Sampling
Type of data	Table
How data was acquired	Collected at random from particular used GSM recharge cards
Data format	Raw
Experimental factors	Test for randomness
Experimental features	Analysis of Variance (ANOVA), Chi-square test of goodness of fit, Chi-square test of independence
Data source location	Covenant University Mathematics Laboratory, Ota, Nigeria.
Data accessibility	All the data are in this data article.

**Value of the data**

• Can be used for educational purposes.
• The method of generation of the data and the data itself will be very helpful in low and middle-income countries where there are little or no computational random number generator for scientific purposes. This is because access to recharge cards is more likely than access to internet in those countries where there is inadequate access to internet to download large datasets caused by either epileptic power supply or lack of internet access. Some of the countries have inadequate internet infrastructure. Even when mobile phones are available, they cannot be compatible with the volume of datasets. See [1]. Also in low and middle-income countries, access to used recharged cards are at no or little cost.
• This research can serve as a cheap way of leveraging on the strong computational algorithms used by GSM companies to generate random datasets. The algorithms are assets through which revenue is accrued through the sale of recharge cards.

## 1 Data

The datasets are the table of random numbers in the raw excel file and the data grouped in four digits in the pdf file. The statistical tests for randomness are indications of the confidence in the reliability of the data for any given purpose.

## 2 Experimental design, materials and methods

Every attempt to construct a random number table must take into account that the table must be independent on any row or column. Furthermore, the data will not be found to follow any observed pattern(s). See [2], [3], [4], [5], [6], [7], [8], [9], [10] for details on other methodologies and results. The choice of using the used recharge cards of GSM network operator was based on the fact that their recharge cards are produced by strong computational algorithms that are programmed to generate random digits and numbers. The steps undertaken to obtain the table of random number datasets are listed below in details.

**Step 1**: A random sample of used recharge cards from a particular GSM network was taken. 380 samples were obtained, each is 16 digits. The number of the digits (0–9) for each of the 16 digits (a–p) is tabulated to show the frequency distribution. This is shown in (Table 1).
**Step 2**: The exploratory data analysis is done to reveal the measures of central tendencies, variation and dispersion of the different columns of the data. This is shown in (Table 2).
**Step 3**: The data were checked for equal or unequal frequency distributions when the digits (0–9) of the 16 columns (a–p) are grouped into mutually exclusive and distinct classes such as; even and odd numbers (Table 3), 0 to 4 and 5 to 9 (Table 4), and 0 and primes against composite numbers (Table 5).
**Step 4**: Analysis of variance (ANOVA) is performed to show the variation between and within the columns (Table 6).
**Step 5**: Correlation among the columns were investigated by computing the Spearman rank correlation coefficients for the pairs of the columns. Randomness is achieved at zero or near zero correlations as shown in (Table 7). The Chi-square test of independence is conducted on each pair of the columns to investigate whether there is association among them. This is shown using the p-values. The bold sections of (Table 8) are indications of association between the columns and hence the probability of randomness is small.
**Step 6**: Chi-square goodness of fit test is conducted to investigate the random distributions of the digits (0–9) shown in (Table 9a, Table 9b). Near zero values of the p-values implies lower probability of randomness.
**Step 7**: Chi-square goodness of fit test is conducted to check the random distribution of the digits (0–9) across the columns (a-p) shown in (Table 10). Higher values of p-values are desirable for randomness irrespective of the values of the Chi-square statistics.
**Step 8**: The residuals obtained in step 7 for all the columns against the digits are tabulated. This is shown in (Table 11).
**Step 9**: Randomness is improved when there are equal distributions of the digits (0–9) in the columns (a–p). This step involves randomly manipulations of the numbers in each column using the table of residuals as a guide. This can be done manually or computationally. For example in column a: randomly remove 0 in 9 places, introduce 1 in 3 places, remove 2 in 13 places and so on. This is repeated for all the other 15 columns. The rationale is to achieve equal representation of digits in random sampling irrespective of the columns.
**Step 10**: Analysis of variance (ANOVA) is performed to show the variation between and within the columns. If step 9 is done correctly, it is expected that the variation between the groups will be zero as shown in (Table 12)
**Step 11**: The Chi-square goodness of fit test is performed to show that the occurrence of the digits are equal in distribution and random. This is shown in (Table 13).
**Step 12**: Correlation among the columns are conducted to verify weak associations among the various columns (Table 14). To show the degree of randomness, Chi-square test of independence is performed to show independence or association between the pairs of the columns. Higher values of p-values are desirable for randomness. It can be seen from (Table 15) that all the p-values are greater than 0.05.
**Step 13**: The final data is 380 by 16 table of random numbers.

## Figures and Tables

**Table 1:** The frequency distribution of the digits of the raw datasets.

	A	b	c	d	e	f	g	h	i	j	k	l	m	n	o	p	**Total**
0	47	47	42	38	30	42	35	31	34	37	35	42	35	37	37	48	617
1	35	37	45	34	46	41	37	34	40	41	47	38	29	43	47	40	634
2	51	41	31	38	35	38	41	41	41	33	42	42	46	48	42	39	649
3	40	34	40	47	35	36	42	40	39	34	41	30	30	40	37	32	597
4	36	41	31	36	42	28	46	42	43	41	37	40	42	33	35	40	613
5	38	35	41	40	39	35	28	51	34	42	44	33	47	32	32	29	600
6	39	36	29	31	34	47	36	30	39	46	40	35	42	37	39	36	596
7	39	39	42	33	43	41	42	46	34	38	37	36	40	42	50	41	643
8	54	38	37	44	36	42	38	34	35	36	36	40	35	37	31	38	611
9	1	32	42	39	40	30	35	31	41	32	21	44	34	31	30	37	520

**Table 2:** The descriptive statistics of the raw datasets.

**Column**	**Mean**	**Median**	**Mode**	**Standard Deviation**	**Skewness**
a	4.05	4	8	2.691	0.023
b	4.32	4	0	2.898	0.042
c	4.47	5	1	2.966	0.011
d	4.51	4	3	2.884	0.029
e	4.57	5	1	2.852	−0.006
f	4.44	5	6	2.892	−0.043
g	4.47	4	4	2.836	0.038
h	4.51	5	5	2.724	0.000
i	4.48	4	4	2.860	0.058
j	4.48	5	6	2.806	−0.041
k	4.21	4	1	2.725	0.088
l	4.51	4	9	2.973	0.006
m	4.56	5	5	2.767	−0.049
n	4.32	4	2	2.859	0.086
o	4.33	4	7	2.843	0.047
p	4.37	4	0	2.969	0.031

**Table 3:** The even and odd number distribution of the raw datasets.

	a	b	c	d	e	f	g	h	i	J	k	l	m	n	o	p	**Total**
Even	227	203	170	187	177	197	196	178	192	193	190	199	200	192	184	201	3086
Odd	153	177	210	193	203	183	184	202	188	187	190	181	180	188	196	179	2994

**Table 4:** The 0–4 and 5–9 distribution of the raw datasets.

	a	b	c	d	e	f	g	h	i	j	k	l	m	n	o	p	**Total**
0−4	209	200	189	193	188	185	201	188	197	186	202	192	182	201	198	199	3110
5−9	171	180	191	187	192	195	179	192	183	194	178	188	198	179	182	181	2970

**Table 5:** The zero prime, and composite numbers distribution of the raw datasets.

	a	b	c	d	e	f	g	h	I	j	k	l	M	n	o	p	**Total**
0 and primes	215	196	196	196	182	192	188	209	182	184	199	183	198	199	198	189	3106
Composite numbers	165	184	184	184	198	188	192	171	198	196	181	197	182	181	182	191`	2974

**Table 6:** The Analysis of Variance of the raw datasets.

**Sources of variation**	**SS**	**df**	**MS**	**F**	**F criteria**	**P value**
Between Groups	110.8710526	15	7.391403509	0.911391394	1.668034532	0.550681054
Within Groups	49179.16842	6064	8.110021178			
Total	49290.03947	6079				

**Table 7:** Correlation coefficients for the raw data sample.

	B	c	d	e	f	g	h	i	j	k	l	m	n	o	p
A	−0.011	0.048	−0.023	0.040	0.076	0.056	−0.057	−0.006	−0.017	−0.028	−0.016	0.015	−0.014	0.009	−0.030
B		0.142	−0.070	−0.012	−0.019	0.050	−0.009	−0.063	−0.014	−0.046	0.087	−0.002	−0.014	0.025	0.001
C			0.015	−0.042	0.029	0.049	0.030	−0.074	0.054	0.038	−0.063	0.047	0.015	0.062	−0.016
D				0.099	−0.004	0.048	0.002	0.036	−0.029	−0.036	0.014	−0.046	−0.030	0.102	−0.054
E					0.045	−0.022	0.127	−0.114	0.024	−0.013	0.052	−0.008	0.045	−0.038	0.055
F						0.083	−0.063	−0.027	−0.028	−0.018	−0.007	0.060	0.060	0.056	0.001
G							−0.027	−0.023	−0.073	−0.016	0.116	−0.041	−0.048	0.099	−0.059
h								−0.087	0.019	−0.035	−0.061	−0.039	0.023	−0.069	0.032
i									0.018	0.010	0.019	0.007	−0.053	0.051	0.067
j										0.032	−0.003	0.015	−0.004	0.004	−0.007
k											0.099	−0.037	−0.090	−0.079	−0.007
l												0.045	0.005	0.043	−0.048
m													−0.097	0.034	−0.076
n														−0.015	−0.086
o															−0.018

**Table 8:** P value of chi-square test of independence for the raw datasets.

	b	c	d	e	f	g	h	i	j	k	l	m	n	o	p
a	0.838	0.808	0.393	0.078	0.111	0.927	0.845	0.776	0.974	0.261	0.483	0.749	**0.016**	0.762	0.918
b		0.411	0.289	0.317	0.327	0.460	0.703	0.412	0.993	0.528	0.304	0.769	0.414	0.761	0.934
c			0.672	0.296	0.925	0.717	0.854	0.849	0.283	0.076	0.953	0.302	0.371	0.740	0.876
d				0.058	0.934	0.091	0.874	0.961	0.409	**0.006**	0.873	0.580	0.948	0.495	0.502
e					0.546	0.763	0.469	0.662	0.865	0.411	0.573	0.493	0.685	0.193	0.145
f						0.967	0.382	**0.03**	0.695	0.313	0.599	0.735	0.446	0.614	0.157
g							0.799	0.310	0.262	0.126	**0.045**	0.229	0.224	0.405	0.486
h								0.448	0.417	0.096	0.178	0.965	0.165	0.086	0.600
i									0.945	0.856	0.235	0.918	0.246	0.336	0.077
j										0.164	0.287	0.553	0.757	0.527	0.635
k											0.279	0.966	0.398	0.936	0.585
l												0.094	0.950	**0.034**	0.772
m													**0.011**	0.601	0.260
n														0.230	0.830
o															0.740

**Table 9a:** Chi-square test of Independence of the raw dataset.

**Digit**	**Observed frequency**	**Expected frequency**	**Residual**
0	617	608	9
1	634	608	26
2	649	608	41
3	597	608	−11
4	613	608	5
5	600	608	−8
6	596	608	−12
7	643	608	35
8	611	608	3
9	520	608	−88

**Table 9b:** Goodness of Fit Test Summary of the raw dataset.

**Test statistics**	**Value**
Chi-square	19.359
df	9
P-value	0.022

**Table 10:** The Goodness of Fit test for all the columns of the raw datasets.

**Column**	**Chi-Square**	**P-value**
a	49.842	0.000
b	4.368	0.886
c	7.632	0.572
d	5.684	0.771
e	5.579	0.781
f	8.105	0.524
g	6.000	0.740
h	12.000	0.213
i	2.789	0.972
j	4.737	0.857
k	11.842	0.222
l	4.684	0.861
m	9.474	0.395
n	6.789	0.659
o	10.579	0.306
p	6.316	0.708

**Table 11:** The Residuals of the Goodness of test of the columns of the raw datasets.

	a	b	c	d	e	f	g	h	i	j	k	l	m	n	o	p	Total
0	9	9	4	0	−8	4	−3	−7	−4	−1	−3	4	−3	−1	−1	10	9
1	−3	−1	7	−4	8	3	−1	−4	2	3	9	0	−9	5	9	2	26
2	13	3	.7	0	−3	0	3	3	3	5	4	4	8	10	4	1	41
3	2	−4	2	9	−3	−2	4	2	1	−4	3	−8	−8	2	−1	−6	−11
4	−2	3	−7	−2	4	−10	8	4	5	3	−1	2	4	−5	−3	2	5
5	0	−3	3	2	1	−3	−10	13	−4	4	6	−5	9	−6	−6	−9	−8
6	1	−2	−9	−7	−4	9	−2	−8	1	8	2	−3	4	−1	1	−2	−12
7	1	1	4	−5	5	3	4	8	−4	0	−1	−2	2	4	12	3	35
8	16	0	−1	6	−2	4	0	−4	−3	−2	−2	2	−3	−1	−7	0	3
9	−37	−6	4	1	2	−8	−3	−7	3	−6	−17	6	−4	−7	−8	−1	−88

**Table 12:** Analysis of Variance of the Final datasets.

**Sources of variation**	**SS**	**df**	**MS**	**F**	**F criteria**	**P value**
Between Groups	0	15	0	0	1.668034532	1.00
Within Groups	50160	6064	8.27176781			
Total	50160	6079				

**Table 13:** The Goodness of Fit test for all the columns of the final datasets.

**Column**	**Chi-Square**	**P-value**
a	0.000	1.000
b	0.000	1.000
c	0.000	1.000
d	0.000	1.000
e	0.000	1.000
f	0.000	1.000
g	0.000	1.000
h	0.000	1.000
i	0.000	1.000
j	0.000	1.000
k	0.000	1.000
l	0.000	1.000
m	0.000	1.000
n	0.000	1.000
o	0.000	1.000
p	0.000	1.000

**Table 14:** The correlation coefficient for the columns of the final datasets.

	b	c	d	e	f	g	h	i	j	k	l	m	n	o	p
a	−0.024	0.009	−0.018	0.070	0.086	0.017	−0.036	0.055	−0.037	−0.023	−0.099	−0.024	0.026	0.000	−0.074
b		0.143	−0.057	−0.001	0.005	0.028	0.018	−0.058	0.011	−0.033	0.061	0.014	−0.072	0.063	0.109
c			0.010	−0.046	0.001	0.005	0.065	−0.060	0.096	−0.010	−0.039	0.071	0.029	0.066	−0.017
d				0.063	−0.036	−0.057	−0.004	0.044	−0.031	−0.028	0.085	0.010	0.000	0.070	−0.037
e					0.004	0.026	0.096	−0.053	0.055	−0.008	0.006	0.042	−0.041	−0.049	−0.010
f						0.022	−0.043	−0.025	0.020	0.056	−0.044	0.074	0.062	0.059	−0.013
g							0.012	−0.052	−0.100	0.039	0.028	−0.110	−0.031	0.034	−0.022
h								−0.070	−0.013	0.018	−0.002	−0.049	−0.005	−0.049	−0.052
i									0.005	−0.006	0.061	−0.042	−0.082	−0.001	0.039
j										0.022	0.021	0.046	0.056	0.047	−0.032
k											0.063	−0.023	0.004	−0.069	−0.104
l												−0.069	0.019	0.067	−0.003
m													0.020	0.033	−0.029
n														−0.026	0.002
o															−0.015

**Table 15:** The P-value for the columns of the final datasets.

	b	c	d	e	f	g	H	I	j	k	l	m	n	o	p
a	0.337	0.527	0.610	0.104	0.803	0.098	0.996	0.978	0.899	0.494	0.721	0.445	0.366	0.791	0.511
b		0.366	0.281	0.351	0.337	0.220	0.777	0.429	0.922	0.735	0.256	0.382	0.721	0.881	0.577
c			0.527	0.511	0.976	0.269	0.957	0.511	0.159	0.861	0.705	0.150	0.577	0.861	0.721
d				0.764	0.735	0.365	0.965	0.957	0.366	0.177	0.097	0.065	0.915	0.477	0.969
e					0.351	0.436	0.118	0.413	0.659	0.750	0.527	0.950	0.561	0.337	0.111
f						0.497	0.544	0.111	0.839	0.308	0.133	0.935	0.643	0.281	0.198
g							0.752	0.285	0.362	0.363	0.215	0.132	0.825	0.952	0.189
h								0.527	0.477	0.413	0.828	0.735	0.413	0.198	0.978
i									0.750	0.544	0.168	0.839	0.627	0.750	0.659
j										0.594	0.850	0.610	0.494	0.544	0.941
k											0.295	0.337	0.231	0.992	0.922
l												0.125	0.735	0.675	0.231
m													0.104	0.168	0.198
n														0.198	0.881
o															0.690

## References

[1] UN News Centre, 〈http://www.un.org/apps/news/story.asp?NewsID=51924#.WAN-f_Sx_cs〉, 2015.

[2] Sharma A.K. (2005). Textbook of Sampling and Attributes.

[3] Tippett L.H.C. (1927). Random Sampling Numbers (41,600), Tracts for Computers, No. 15.

[4] Bennett D.J. (2009). Randomness. http://https://books.google.com.ng/books?id=CdgPEhJh5tUC&dq=tippett+1927&source=gbs_navlinks_s.

[5] R.A. Fisher, F. Yates, Statistical tables for biological, agricultural and medical research 3rd ed., 1938, pp. 26–27. ISBN: 0-02-844720-4.

[6] M.G.Kendall and B.B.SmithRandomness and Random Sampling numbers, J. Roy. Statist. Soc., 101, 1, pp. 147–166. (1938).

[7] 〈http://www.rand.org/pubs/monograph_reports/MR1418.html〉

[8] Hull T.E., Dohell A.R. (1962). Random numbers generators. SIAM Rev..

[9] Yule G.U. (1938). A test of Tippett׳s random sampling numbers. J. R. Stat. Soc..

[10] Kirkpatrick S., Stoll E.P. (1981). A very fast shift-register sequence random number generator. J. Comput. Phys..

