# Supplementary material for: Random number datasets generated from statistical analysis of randomly sampled GSM recharge cards
Source: Data Brief. 2016 Dec 9;10:269–76. doi: 10.1016/j.dib.2016.12.003 (PMC5167235; doi:10.1016/j.dib.2016.12.003)
Supplement: Supplementary file 1 — Supplementary material [file mmc1.docx]

CONFLICT OF INTEREST FORM

The authors acknowledged the absence of conflicts of interest. The data has not been published elsewhere and all the contributions were duly acknowledged.

Plagiarism test can attest to the claim(s).
