# Supplementary material for: Random number datasets generated from statistical analysis of randomly sampled GSM recharge cards
Source: Data Brief. 2016 Dec 9;10:269–76. doi: 10.1016/j.dib.2016.12.003 (PMC5167235; doi:10.1016/j.dib.2016.12.003)
Supplement: Supplementary file 2 — Supplementary material [file mmc2.zip › mmc2/Supplementary DATA 2.docx]

6105 5278 3106 4623

5302 4310 3279 2659

9211 7539 3811 1744

6334 9369 6442 0437

4679 5872 9100 8155

6962 2248 1234 5619

1419 8756 6378 7878

6654 4884 9893 3769

2748 5079 7047 2264

7477 7801 5601 9977

5389 2463 1415 4536

3428 7639 6166 1000

2608 6526 0879 8469

3138 9306 7122 9944

8595 4864 1930 6176

1690 1336 8366 7635

2143 7534 3704 9820

0719 4007 9659 4759

8572 3392 6048 5224

7525 8178 5480 1031

6187 0862 9053 2628

1099 7969 4979 7394

6948 4108 6824 0959

4761 5991 1307 6716

5291 1315 9853 5688

9123 0271 1266 4824

6328 5860 7775 3673

2692 9687 2319 1686

3479 8735 5126 8472

1306 5496 0282 5747

4273 1289 1394 7110

5559 0061 1063 4261

2568 9535 2774 2916

4484 6524 7938 3464

8044 4891 4492 8538

9669 3105 5357 0085

6595 9297 8918 7308

4952 8791 9539 5683

9340 1008 6022 9065

2159 0445 5155 1924

4052 6022 4916 2453

7472 3698 0674 4952

9288 7621 7501 5267

0755 9979 4097 9070

3927 5095 0439 7198

2501 0729 5653 4929

8878 8844 4835 3236

1103 5617 5410 9512

1269 4420 8102 5245

7982 3931 4659 1196

3500 1190 6123 0017

3490 7464 0470 3603

7653 6035 8942 4681

6583 2568 9728 3748

5146 6687 5237 0439

3168 7357 4181 4222

9073 1745 3940 7686

2648 2481 9398 5069

0977 3469 0983 4553

9323 2008 6505 3165

0016 0774 5113 0368

8094 1093 8869 7533

3102 6891 5844 0373

3609 4214 4687 6852

3676 9181 5219 7573

5598 5950 1965 3092

1985 6105 7484 2214

4757 7290 2573 6472

9676 7641 0198 0958

1667 6874 6456 8187

7885 1737 2680 4639

1111 6901 9213 6345

3319 2414 3464 2737

6585 4663 0692 9808

2210 9295 7881 5493

1166 8338 2340 4349

4756 3789 7238 7405

7118 4020 1717 6224

3439 2378 8649 3931

2345 2252 6702 8288

7087 3494 5356 2890

2926 4910 6913 1602

0897 7098 4035 6139

6110 6046 8701 5797

0176 2105 6887 9925

1621 6459 8522 7408

9997 1590 0104 9963

0301 7782 4596 6300

4984 8230 2342 2051

0775 2976 9165 0488

6146 8343 7587 8774

1349 9127 2663 6215

2260 3383 8982 1382

8368 9251 5324 3916

8022 8916 8246 4514

0255 1869 0401 9752

4371 6252 2069 8119

6707 5389 4108 0366

9809 7170 5225 9210

7362 9499 6133 2519

0419 6648 1874 7634

2994 8350 6126 9855

0436 2067 3135 0422

7661 2755 0203 8277

6253 3586 5511 5700

8538 5811 8392 2114

9286 9228 5828 0841

9174 7714 4160 7652

5543 2688 2863 8088

2953 8372 1944 4527

9572 7902 8871 8865

7427 3244 7038 3242

0114 1132 5351 8706

7007 9623 3212 9355

8570 3670 2501 7997

9056 2413 8310 8110

1254 6537 0797 2006

8581 7343 9556 6435

9108 9466 8168 7951

4009 2973 2029 1608

2499 1829 7786 3783

9034 0201 8231 0537

8843 8450 0314 7895

4371 3017 9063 5004

6823 7165 2505 9282

9814 5030 1610 1376

2018 7704 4932 0731

3006 4688 7468 3974

5265 1644 9651 5352

1168 6010 4919 8135

7853 8878 4125 1367

4552 4467 6767 0796

0088 0702 6031 2463

1218 3025 3908 8578

3051 1065 2516 5685

9007 7579 1130 7811

6375 5723 4407 5997

7752 2956 3256 4268

3582 0970 3475 6312

5150 9197 0291 5013

2757 4669 9678 0171

3815 1253 1949 3239

4468 5633 0356 8592

3651 7265 2108 1620

5015 1551 6055 4754

0923 4642 8347 2347

5739 7230 9098 5548

3964 5129 3369 3861

7403 0570 2054 8456

9753 2512 7072 7837

6052 5963 4283 6738

3237 5426 7214 4225

8815 3710 9943 0048

5124 4547 4651 2601

6771 2382 3567 0812

5934 9656 7719 6154

3446 1962 6208 9685

6024 8600 2466 2547

5637 0673 8474 6729

3088 1316 2893 7842

5001 3052 2215 0921

7872 0810 7377 6386

5226 6393 7503 1644

8343 6852 0331 9534

7241 5576 7400 4076

5104 3440 8637 0280

8468 8053 8458 1101

7433 1173 5184 5715

6982 0725 1461 8692

1337 7869 3688 6563

0972 9403 5515 9097

1204 3951 0720 0424

8894 0275 5673 3705

3720 4381 5181 6656

4731 5920 3865 0570

9563 6092 1248 2984

7697 7576 9915 9276

0224 9301 5376 6789

5983 1848 0084 4483

4890 7971 3565 0631

8828 1729 3282 3164

4019 7583 2134 5381

2221 9190 7793 5945

5852 7457 0961 6008

1052 7025 2689 3826

8770 8781 4432 9454

9017 9334 6598 2586

2932 1312 9054 5271

1820 7168 7541 7716

1227 2573 3128 4325

Kirkpatrick, S., & Stoll, E. P. (1981). A very fast shift-register sequence random number generator. *Journal of Computational Physics*, *40*(2), 517-526.

- [View at Publisher](https://www.scopus.com/redirect/linking.uri?targetURL=http%3a%2f%2fdx.doi.org%2f10.1007%2f978-3-319-15350-6_12&locationID=1&categoryID=4&eid=2-s2.0-84922365478&issn=03029743&linkType=ViewAtPublisher&year=2015&origin=recordpage&dig=2c8c369f4501c006bb633c35c01cef68&recordRank=)|

Top of Form

[Text export](https://www.scopus.com/record/display.uri?eid=2-s2.0-84922365478&origin=resultslist&sort=plf-f&src=s&st1=pseudorandom+number+table&st2=&sid=B257266422E5D00712B71A0847F15510.aXczxbyuHHiXgaIW6Ho7g%3a321&sot=b&sdt=b&sl=40&s=TITLE-ABS-KEY%28pseudorandom+number+table%29&relpos=0&citeCnt=0&searchTerm=)

Bottom of Form

- | [Download](https://www.scopus.com/record/display.uri?eid=2-s2.0-84922365478&origin=resultslist&sort=plf-f&src=s&st1=pseudorandom+number+table&st2=&sid=B257266422E5D00712B71A0847F15510.aXczxbyuHHiXgaIW6Ho7g%3a321&sot=b&sdt=b&sl=40&s=TITLE-ABS-KEY%28pseudorandom+number+table%29&relpos=0&citeCnt=0&searchTerm=)
- | [Save to list](https://www.scopus.com/record/display.uri?eid=2-s2.0-84922365478&origin=resultslist&sort=plf-f&src=s&st1=pseudorandom+number+table&st2=&sid=B257266422E5D00712B71A0847F15510.aXczxbyuHHiXgaIW6Ho7g%3a321&sot=b&sdt=b&sl=40&s=TITLE-ABS-KEY%28pseudorandom+number+table%29&relpos=0&citeCnt=0&searchTerm=)
- | [More...](https://www.scopus.com/record/display.uri?eid=2-s2.0-84922365478&origin=resultslist&sort=plf-f&src=s&st1=pseudorandom+number+table&st2=&sid=B257266422E5D00712B71A0847F15510.aXczxbyuHHiXgaIW6Ho7g%3a321&sot=b&sdt=b&sl=40&s=TITLE-ABS-KEY%28pseudorandom+number+table%29&relpos=0&citeCnt=0&searchTerm=)

[Lecture Notes in Computer Science (including subseries Lecture Notes in Artificial Intelligence and Lecture Notes in Bioinformatics)](https://www.scopus.com/source/sourceInfo.uri?sourceId=25674&origin=recordpage)

Volume 8904, 2015, Pages 188-203

6th TPC Technology Conference on Performance Evaluation and Benchmarking, TPCTC 2014 held in conjunction with 40th International Conference on Very Large Data Bases, VLDB 2014; Hangzhou; China; 1 September 2014 through 5 September 2014; Code 113699

**Composite key generation on a shared-nothing architecture  (Conference Paper)**

[Hoffmann, M.](https://www.scopus.com/authid/detail.uri?authorId=56506280900&amp;eid=2-s2.0-84922365478)[^a^](https://www.scopus.com/record/display.uri?eid=2-s2.0-84922365478&origin=resultslist&sort=plf-f&src=s&st1=pseudorandom+number+table&st2=&sid=B257266422E5D00712B71A0847F15510.aXczxbyuHHiXgaIW6Ho7g%3a321&sot=b&sdt=b&sl=40&s=TITLE-ABS-KEY%28pseudorandom+number+table%29&relpos=0&citeCnt=0&searchTerm=)

,

[Alexandrov, A.](https://www.scopus.com/authid/detail.uri?authorId=55025823300&amp;eid=2-s2.0-84922365478)[^a^](https://www.scopus.com/record/display.uri?eid=2-s2.0-84922365478&origin=resultslist&sort=plf-f&src=s&st1=pseudorandom+number+table&st2=&sid=B257266422E5D00712B71A0847F15510.aXczxbyuHHiXgaIW6Ho7g%3a321&sot=b&sdt=b&sl=40&s=TITLE-ABS-KEY%28pseudorandom+number+table%29&relpos=0&citeCnt=0&searchTerm=),

[Andritsos, P.](https://www.scopus.com/authid/detail.uri?authorId=6506919220&amp;eid=2-s2.0-84922365478)[^b^](https://www.scopus.com/record/display.uri?eid=2-s2.0-84922365478&origin=resultslist&sort=plf-f&src=s&st1=pseudorandom+number+table&st2=&sid=B257266422E5D00712B71A0847F15510.aXczxbyuHHiXgaIW6Ho7g%3a321&sot=b&sdt=b&sl=40&s=TITLE-ABS-KEY%28pseudorandom+number+table%29&relpos=0&citeCnt=0&searchTerm=),

[Soto, J.](https://www.scopus.com/authid/detail.uri?authorId=7201507433&amp;eid=2-s2.0-84922365478)[^a^](https://www.scopus.com/record/display.uri?eid=2-s2.0-84922365478&origin=resultslist&sort=plf-f&src=s&st1=pseudorandom+number+table&st2=&sid=B257266422E5D00712B71A0847F15510.aXczxbyuHHiXgaIW6Ho7g%3a321&sot=b&sdt=b&sl=40&s=TITLE-ABS-KEY%28pseudorandom+number+table%29&relpos=0&citeCnt=0&searchTerm=),

[Markl, V.](https://www.scopus.com/authid/detail.uri?authorId=6602853794&amp;eid=2-s2.0-84922365478)[^a^](https://www.scopus.com/record/display.uri?eid=2-s2.0-84922365478&origin=resultslist&sort=plf-f&src=s&st1=pseudorandom+number+table&st2=&sid=B257266422E5D00712B71A0847F15510.aXczxbyuHHiXgaIW6Ho7g%3a321&sot=b&sdt=b&sl=40&s=TITLE-ABS-KEY%28pseudorandom+number+table%29&relpos=0&citeCnt=0&searchTerm=)

^a^ DIMA, Technische Universität Berlin, Einsteinufer 17, Berlin, Germany
^b^ Institut des Systémes d’Information, Universit´e de Lausanne, Bâtiment Internef, 1015 Lausanne, Switzerland

[View references (11)](https://www.scopus.com/record/display.uri?eid=2-s2.0-84922365478&origin=resultslist&sort=plf-f&src=s&st1=pseudorandom+number+table&st2=&sid=B257266422E5D00712B71A0847F15510.aXczxbyuHHiXgaIW6Ho7g%3a321&sot=b&sdt=b&sl=40&s=TITLE-ABS-KEY%28pseudorandom+number+table%29&relpos=0&citeCnt=0&searchTerm=#references)

**Abstract**

Generating synthetic data sets is integral to benchmarking, debugging, and simulating future scenarios. As data sets become larger, real data characteristics thereby become necessary for the success of new algorithms. Recently introduced software systems allow for synthetic data generation that is truly parallel. These systems use fast pseudorandom number generators and can handle complex schemas and uniqueness constraints on single attributes. Uniqueness is essential for forming keys, which identify single entries in a database instance. The uniqueness property is usually guaranteed by sampling from a uniform distribution and adjusting the sample size to the output size of the table such that there are no collisions. However, when it comes to real composite keys, where only the combination of the key attribute has the uniqueness property, a different strategy needs to be employed. In this paper, we present a novel approach on how to generate composite keys within a parallel data generation framework. We compute a joint probability distribution that incorporates the distributions of the key attributes and use the unique sequence positions of entries to address distinct values in the key domain. © Springer International Publishing Switzerland 2015.

**Indexed keywords**

**Engineering controlled terms:** Benchmarking; Number theory; Probability distributions; Random number generation

RAND CORPORATION

http://www.rand.org/pubs/monograph_reports/MR1418.html
